# Temperature and Impedance Variations During Tumor Treating Fields (TTFields) Treatment

**DOI:** 10.3389/fnhum.2022.931818

**Published:** 2022-07-11

**Authors:** Nichal Gentilal, Eyal Abend, Ariel Naveh, Tal Marciano, Igal Balin, Yevgeniy Telepinsky, Pedro Cavaleiro Miranda

**Affiliations:** ^1^Instituto de Biofísica e Engenharia Biomédica, Faculdade de Ciências da Universidade de Lisboa, Campo Grande, Lisbon, Portugal; ^2^Novocure Ltd, Haifa, Israel

**Keywords:** finite element method (FEM), glioblastoma multiforme (GBM), head impedance, realistic head model, tissue heating, NovoTAL system, Tumor Treating Fields (TTFields)

## Abstract

Tumor Treating Fields (TTFields) is an FDA-approved cancer treatment technique used for glioblastoma multiforme (GBM). It consists in the application of alternating (100–500 kHz) and low-intensity (1–3 V/cm) electric fields (EFs) to interfere with the mitotic process of tumoral cells. In patients, these fields are applied via transducer arrays strategically positioned on the scalp using the NovoTAL™ system. It is recommended that the patient stays under the application of these fields for as long as possible. Inevitably, the temperature of the scalp increases because of the Joule effect, and it will remain above basal values for most part of the day. Furthermore, it is also known that the impedance of the head changes throughout treatment and that it might also play a role in the temperature variations. The goals of this work were to investigate how to realistically account for these increases and to quantify their impact in the choice of optimal arrays positions using a realistic head model with arrays positions obtained through NovoTAL™. We also studied the impedance variations based on the log files of patients who participated in the EF-14 clinical trial. Our computational results indicated that the layouts in which the arrays were very close to each other led to the appearance of a temperature hotspot that limited how much current could be injected which could consequently reduce treatment efficacy. Based on these data, we suggest that the arrays should be placed at least 1 cm apart from each other. The analysis of the impedance showed that the variations seen during treatment could be explained by three main factors: slow and long-term variations, array placement, and circadian rhythm. Our work indicates that both the temperature and impedance variations should be accounted for to improve the accuracy of computational results when investigating TTFields.

## Introduction

Glioblastoma multiforme (GBM) is one of the deadliest tumors that appears in the brain. It is ranked by the World Health Organization (WHO) as a grade IV glioma, which is the highest and therefore the most dangerous classification assigned to a central nervous system disease (Louis et al., 2016). Despite the recent advances in medicine, the etiology of GBM is still not known and the prognosis remains very poor. The median survival rate is between 14.6 and 16.7 months from diagnosis (Stupp et al., 2012) and the five-year survival rate is only 5% (Ostrom et al., 2017). Even in the cases where first line GBM treatment is apparently successful, the probability of reoccurrence is very high and thus, very often, treatment is performed with a palliative intent. For several years, surgery, radiation therapy and chemotherapy were the most used techniques and were the standard of care for glioblastoma. However, the study performed by Kirson et al. (2004) in 2004 showed the potential of also using electric fields (EFs) as an additional line of treatment.

The application of these fields as a cancer treatment technique was named Tumor Treating Fields (TTFields), and it consists in the application of an EF with a frequency between 100 and 500 kHz. *In-vitro* studies showed that these EFs can arrest tumoral cells proliferation for intensities above 1 V/cm at the tumor bed (Kirson et al., 2004). The mechanisms of action are still not completely understood but the first hypotheses suggested an interference with the mitotic process in two different stages (Kirson et al., 2004, 2007). The first occurs at the early stages of mitosis and it consists in the inhibition and/or prolongation of cell division when tubulin polymerization-depolymerization drives the proliferation process. At this stage, the induced EF may orientate tubulin dimers according to its own direction due to their large intrinsic dipole moment. As these dimers are one of the major components of the microtubules, the mitotic spindle is not able to form properly and thus cannot correctly align and separate chromosomes. The second mechanism occurs during cytokinesis when the dividing cell acquires an hourglass morphology. The application of an external EF gives rise to a non-uniform intracellular field, with a high density at the cleavage furrow. Due to a physical phenomenon known as dielectrophoresis, polarizable macromolecules and ions contained in the cell are pulled toward the furrow, regardless of the field polarity. An accumulation of these components leads to membrane blebbing and consequently cell destruction. Since this technique was first reported, other mechanisms of action were also studied and suggested (Gera et al., 2015; Giladi et al., 2017; Chang et al., 2018; Berkelmann et al., 2019; Rominiyi et al., 2021).

TTFields are FDA-approved as a monotherapy for the treatment of recurrent GBM cases since 2011, following the promising results of the EF-11 clinical trial (Stupp et al., 2012). Three years later, in 2014, this technique was also approved as an adjuvant therapy to chemotherapy with temozolomide (TMZ) for the treatment of newly diagnosed GBM cases after the results of the EF-14 clinical trial (Stupp et al., 2017). Nowadays, TTFields are administrated to patients using a specific device named Optune that was created by Novocure. This device, formerly known as NovoTTF-100A, consists of an electric field generator connected to four transducer arrays that work in pairs. Each array is strategically placed on the patient's shaved scalp, and it consists in a 3 x 3 matrix of transducers whose centers are separated by 22 mm in one direction and 44 mm in the other. Between the transducers and the scalp, there is a thin layer of conductive hydrogel that optimizes electric field coupling to the head. Current is injected in two perpendicular directions alternately with a switching time of one second as application of the EFs in more than one direction was shown to increase the number of cells affected by this technique (Kirson et al., 2004). It is important to emphasize that there is no charge exchange between the device and the patient and thus the term current injection is used for simplicity. The regions where the arrays are placed are defined by the Novo Transducer Array Layout (NovoTAL™) system. This software measures the dimensions of the head in specific regions and suggests which layouts might be the most appropriate for treatment (Chaudhry et al., 2015). In the literature, the effectiveness of each layout is typically quantified by injecting 900 mA of current into each pair of arrays. The best treatment option is considered to be the one that yields the highest average EF value in the tumor.

*Post-hoc* analyses of data from the EF-11 and EF-14 clinical trials allowed to improve how this technique was applied to enhance treatment outcomes. Based on the studies by Kanner et al. (2014) and Toms et al. (2019), the fields should be applied for at least 28 consecutive days and the time that the patient is under treatment, i.e., the daily usage, should be maximized. According to Kanner et al. (2014), the overall survival in the group of patients whose daily treatment time was at least 18 h was significantly higher than in the complementary group. The main adverse event that occurred due to treatment was skin reaction, which affected around 25% of patients (Mrugala et al., 2014). Its cause was attributed to the use of the hydrogel, but it was easily treated with topical corticosteroids, by slightly shifting the arrays, or by stopping treatment for a few days. The second most common adverse event was a heat sensation felt by 11% of patients (Mrugala et al., 2014). The application of the EFs heats up the head as a result of the Joule effect, and the temperature remains above basal values for long periods of time due to the high daily usage needed.

To avoid any thermal harm to the patient, scalp's temperature is monitored underneath each transducer and the current is limited so as to keep the maximum temperature on the scalp below 39.5 °C during treatment. The minimum current amplitude that is injected is 400 mA, and the maximum is 1,000 mA. Thus, the 900 mA that are typically considered in the literature are at the high-end of this range. Some studies quantified treatment efficacy (e.g.: Miranda et al., 2014; Wenger et al., 2015) and investigated the best array layout as a function of tumor positioning (e.g., Wenger et al., 2016; Korshoej et al., 2018) through *in-silico* work in which this specific amount of current was injected. As it might not always be possible to inject the 900 mA due to the thermal restrictions of the therapy, the EF in the tumor might not reach the threshold of 1 V/cm above which TTFields are most effective in disrupting cell division (Kirson et al., 2004).

The temperature variations that occur during TTFields therapy were first investigated by Gentilal et al. (2019) through *in-silico* studies. In that work, it was seen that, in each tissue, the temperature increased mainly underneath the regions where the arrays were placed, and they were very superficial. These conclusions were drawn by injecting 900 mA of current into each array pair, which might also not mimic the current injected in patients due to the reasons discussed above. The goals of this work were: (1) study how to add information about the current injected into each pair based on Optune's current injection algorithm; (2) quantify its relevance on the predicted treatment effectiveness and on the choice of the best layout using different NovoTAL layouts; (3) suggest ways to indirectly account for the temperature increases to optimize treatment planning; and (4) investigate how the impedance varies during treatment and discuss how it might affect the previous conclusions.

## Methods

### Realistic Head Models

The realistic head model used in this work was built based on MR images available for the single-subject template Colin27 and it was the same one used in the first studies on heat transfer during TTFields therapy (Gentilal et al., 2019, 2020; Gentilal and Miranda, 2020). Based on the T1 and proton density images made available by Brainweb (https://brainweb.bic.mni.mcgill.ca/), the relevant tissues were segmented using Brainsuite, as described in detail in Miranda et al. (2013). The cerebrospinal fluid (CSF) and the brain, divided into gray matter (GM) and white matter (WM), were segmented using the T1 images, whereas the scalp and the skull were segmented using the proton density images. In Mimics, small irregularities were corrected, and additional anatomical detail was added manually. More specifically, the lateral ventricles were segmented by thresholding the data of the WM. At this point, a spherical virtual lesion was also added to the model to mimic a glioblastoma (Miranda et al., 2014). This lesion consisted in a spherical necrotic core, with a radius of 7 mm, surrounded by a concentric active shell, with a radius of 10 mm. It was placed in the right hemisphere of the brain near the ventricles and at about the same distance to the anterior and posterior regions of the head.

Then, we used the NovoTAL system to create different array layouts for our model. Each one of the five layouts built was composed by two pairs, referred to as anterior-posterior (AP) and left-right (LR). Between each transducer and the scalp, a thin layer of gel was added in Mimics. The average thickness of this layer was around 0.7 mm, which is very close to 0.6 mm of gel that already comes embedded in the arrays (Hershkovich et al., 2019).

### Predicting the Electric Field Distribution

At the frequency at which the Optune device operates to treat GBM, 200 kHz, the electroquasistatic approximation of Maxwell's equations is valid (Haus and Melcher, 1989). Under these conditions, the electric field distribution in the head can be obtained solving Laplace's equation twice, one for each pair. This equation is given by:


(1) $$\begin{array}{l} \nabla \cdot \left(\sigma^{{\;}^{*}}\;\nabla \;\phi \right)=0 \end{array}$$

where σ^*^ is the complex electric conductivity (S/m), and ϕ the electrostatic scalar potential (V). The first is given by:


(2) $$\begin{array}{l} \sigma^{*}=\sigma +j\epsilon_{r}\epsilon_{0}\omega \end{array}$$

where σ is the scalar electric conductivity (S/m), *j* the imaginary unit, ϵ_*r*_ the relative permittivity (unitless), ϵ_0_ the permittivity of free space (≈8.854 × 10^−12^ *F*/*m*), and ω the angular frequency (rad/s). This equation reflects the existence of a resistive and a capacitive components of the current density, given by the first and second terms on the right-hand side, respectively. For the frequency at which TTFields are used, the current density in the head is mainly resistive as σ≫*jϵ*_*r*_ϵ_0_ω for all tissues. The values of the dielectric properties of every tissue and material are provided in Table 1.

**Table 1:** Values assigned to the physical properties of each tissue and material.

**Physical parameter**	**Scalp**	**Skull**	**CSF**	**GM**	**WM**	**Tumor active shell**	**Tumor necrotic core**	**Gel**	**Transducers**
Electric conductivity σ (S/m)	0.30	0.08	1.79	0.25	0.12	0.24	1.00	0.10	0
Relative permittivity ϵ_*r*_ (1)	5,000	200	110	3,000	2,000	2,000	110	100	10,000
Thermal conductivity *k* [W/(m °C)]	0.34	1.16	0.60	0.565	0.503	0.550	0.550	0.60	6
Specific heat c [J/(kg °C)]	3,150	1,700	4,200	3,680	3,600	3,600	3,600	4,186	527
Density ρ (kg/m3)	1,000	1,500	1,000	1,036	1,027	1,030	1,030	1,000	6,060
Blood perfusion rate ω^*^(×10^−3^ 1/*s*)	1.43	0.143	0	13.30	3.70	1.72	0	NA	NA
Metabolic rate *Q*_*m*_ (W/m3)	363	70	0	16,229	4,518	58,000	0	NA	NA

At the boundaries, we set the same conditions as in our previous TTFields studies (Miranda et al., 2014; Wenger et al., 2015, 2016; Gentilal et al., 2019, 2020; Gentilal and Miranda, 2020). As each array can be seen as an isopotential surface, a Dirichlet boundary condition was imposed on the outer surfaces of all transducers of the same array:


(3) $$\begin{array}{l} \phi =V_{0} \end{array}$$

where the value of *V*_0_ (V) depends on how much current is injected into each pair. This value was different for each pair of arrays and each layout, and it was chosen following the methodology described in Subsection How to Account for the Temperature.

At the remaining outer boundaries, namely on the scalp, gel and lateral boundaries of the transducers, the normal component of the current density is null. Thus, a Neumann boundary was used:


(4) $$\begin{array}{l} \vec{n}\cdot \vec{J}=0 \end{array}$$

in which $\vec{n}$ is the normal to the surface and $\vec{J}$ is the current density (A/m^2^). This ensured that the head was electrically insulated.

At the internal boundaries, continuity of the normal component of the current density was imposed:


(5) $$\begin{array}{l} \vec{n_{2}}\cdot \left(\vec{J_{2}}\;-\vec{J_{1}}\right)=0 \end{array}$$

where $\vec{n_{2}}$ is the outward normal in medium 2, and current is flowing from medium 1 to medium 2.

### Predicting the Temperature Distribution

In computational studies, the temperature variation is typically investigated and predicted using Pennes' equation (Pennes, 1948). When TTFields are applied, an additional term, *Q*_*JH*_, must be added to this equation to account for Joule heating:


(6) $$\begin{array}{l} \rho \;c\frac{\partial T}{\partial t}=\nabla \cdot \left(k\nabla T\right)+{Q_{met}+Q}_{blood}+Q_{JH} \end{array}$$

In the previous expression ρ represents the density (kg/m3), *c* the specific heat [J/(kg °C)], *T* the temperature (°C), *t* the time (s), and *k* the thermal conductivity [W/(m °C)]. The first term on the right-hand side is known as the Fourier's law for thermal conduction and it accounts for the heat that is transferred through that process. The second term, *Q*_*met*_ (W/m3), represents the heat generated because of the metabolic activity of tissues. In the simulations performed, this term was considered to be constant. The third term, *Q*_*blood*_ (W/m3), expresses the energy exchanges with the blood. It is given by:


(7) $$\begin{array}{l} Q_{blood}=\omega^{*}\rho_{b}c_{b}\left(T_{b}-T\right) \end{array}$$

where ω^*^ is the blood perfusion rate (1/s) and the subscript “b” stands for blood. This equation implies that when the temperature of tissues is below *T*_*b*_, the blood acts like a heat source, whereas when it is above it acts like a heat sink. The value assumed for *T*_*b*_ in the simulations was 36.7°C.

The Joule heating term, *Q*_*JH*_ (W/m3), represents the contribution of the EFs in increasing the temperature of tissues. Under the electroquasistatic approximation, this term is given by:


(8) $$\begin{array}{l} Q_{JH}=\sigma ||\vec{E}{||}^{2} \end{array}$$

in which $\vec{E}$ is the electric field vector (V/m). The contribution of each pair was taken alternately with a switching time of 1 s.

At the outer boundaries, two additional mechanisms were considered. Convection is mathematically described by:


(9) $$\begin{array}{l} F_{conv}=h\left(T_{surface}-T_{room}\right) \end{array}$$

where *h* is the convection factor [W/(m^2^ °C)], and *T*_*surface*_ is the temperature of the surface (°C) that is in contact with the environment at a temperature *T*_*room*_(°C).

Radiation is given by Stefan-Boltzmann's law:


(10) $$\begin{array}{l} F_{rad}=\epsilon \sigma_{SB}\left({\left(T_{surface}+273.15\right)}^{4}-{\left(T_{room}+273.15\right)}^{4}\right) \end{array}$$

where ϵ is the emissivity factor (unitless) and σ_*SB*_ is the Stefan-Boltzmann constant [≈5.668 × 10^−8^ W/(m^2^ °*C*^4^)]. Both *F*_*conv*_ and *F*_*rad*_ are in W/m^2^. The value of *T*_*room*_ was assumed to be 24°C in the simulations.

At the internal boundaries, it was assumed that there was conduction between adjacent tissues and materials.

The values assigned to the physical parameters are presented in Table 1. The thermal ones were the standard values presented in the sensitivity analysis performed by Gentilal and Miranda (2020), and they were taken either from Hasgall et al. (2018) or from Duck (2013). The electric properties were taken from Ballo et al. (2019) as the dataset that we used for the impedance analysis (described in detail in Subsection Limitations of the Approach Used: A Preliminary Investigation Based on the Impedance) was the same one as in that work. The convection factor of all outer boundaries was assumed to be 4 W/(m^2^ °C) and the emissivity was considered to be 1. These correspond to the values that are typically considered for the skin (Gentilal and Miranda, 2020). Blood density and specific heat were 1,050 kg/m3 and 3,600 J/(kg °C), respectively (Gentilal and Miranda, 2020). We assumed that all properties were isotropic and uniform.

### How to Account for the Temperature

As discussed before, due to the thermal restrictions of the therapy, the amount of current injected into each pair might not be exactly 900 mA, and it does not have to be the same for both pairs. We modeled Optune's current injection algorithm using a simplified head model to predict how much current could be injected into each pair if the temperature was also accounted for. However, the results indicated that it would take several weeks to obtain useful results from which conclusions could be drawn. Thus, an alternative approach had to be sought that could still maintain the accuracy of the results, but that could also be computed faster.

We started by investigating the information available in the log files of one patient treated with TTFields. These files are data stored in Optune's memory bank and which contain information about the voltage, current, and impedance throughout therapy, as well as the duration of treatment (Hershkovich et al., 2019). The impedance registered by the device is measured between the arrays and it is a result of the contribution of the impedance of the head, of the transducers, and of the contact impedance at the gel-scalp boundary. The temperature recorded by each thermistor is also logged in regularly, among other data.

Based on this information and comparing it with *in-silico* data, we concluded that the amount of current that leads to a maximum steady-state temperature of 39.5°C on the scalp in the model was a good indicator of the average current that was injected during treatment. For the electric parameters presented in Table 1, the impedance of the model was also close to what was seen in the log files (around 60 Ω).

As discussed by Gentilal et al. (2021), when TTFields are applied the temperature of this tissue tends exponentially to a new steady state and it can be modeled by the following general expression:


(11) $$\begin{array}{l} T=C_{1}\left(1-exp\;\left(-t/C_{2}\right)\right)+C_{3} \end{array}$$

where *C*_1_ (°C), *C*_2_ (s), and *C*_3_ (°C) are fitted coefficients. The first represents the contribution of the EFs in increasing the temperature of the scalp and it depends on how much current is injected. The second is a time constant representative of how quickly scalps heats up. The third represents the temperature of the scalp before current injection starts. This equation is valid assuming that the head is in thermal equilibrium with the environment before current injection.

The maximum steady-state temperature can be predicted as:


(12) $$\begin{array}{l} T^{\max}=\underset{t\to \infty }{lim}\;T=C_{1}+C_{3} \end{array}$$

Curve fitting was performed in Matlab 2020a using the appropriate toolbox (cftool).

Thus, to predict the average EF intensity in the tumor accounting for the temperature variations, it is necessary to iteratively investigate how much current can be injected into each pair to induce a maximum steady-state temperature, *T*^max^, of 39.5 °C on the scalp underneath each pair.

As discussed in Miranda et al. (2014), in this head model the AP pair is the one that contributes the least to treatment due to the tumor's location assuming constant and equal current in both pairs. Thus, the criterion used in this work to choose how much current was injected into each pair was to first maximize the current injected into the AP pair and only then in the LR pair. This means that the current that leads to 39.5 °C on the scalp underneath the former pair was investigated first and after that value was found and fixed, the current that leads to the same temperature value on the scalp, or as close as possible to it, was determined for the LR pair. These values do not have to be the same for both pairs, but they are bounded by 400 and 1,000 mA, which is the same range considered in the Optune system.

All simulations were done in COMSOL Multiphysics v.5.2a in a workstation with a dual core Intel Core i9-10900X X-series processors clocked at 3.7 GHz and 64 GB of RAM. Each electric simulation took around 2 h to compute, whereas it took around 40 h to simulate the temperature variations for the first 5 min of treatment.

### Limitations of the Approach Used: A Preliminary Investigation Based on the Impedance

The approach described in the previous section to predict how much current could be injected into each pair was based on the comparison of *in-silico* data with the values seen in the log files of one patient treated with TTFields. However, there can be a high inter-subject variability of the information available in these files. In this work, we did a preliminary study in which we investigated the sensitivity of some of the most relevant data that could be retrieved from these log files, and we discuss possible limitations of the approach that we followed in the previous section.

One way to validate the model is by comparing its impedance with what is seen in the log files. This can help to fine-tune the electric parameters and to ensure that the EF distribution and the Joule effect in the model are accurately estimated.

To investigate the inter-subject variability of the impedance, log files from the devices of 340 patients who participated in EF-14 trial and that were also included in the analysis performed by Ballo et al. (2019) were considered. On average, patients were under treatment 12 months (standard deviation: 9.5 months). During that time, Optune measured and recorded the voltages and currents applied to each channel (AP and LR), saving logs every 30 min, indicating the values measured at that timepoint. The Lomb-Scargle method (Scargle, 1982) was used to study the variations on the impedance values. This method allows to estimate the frequency spectrum of unevenly spaced signal by fitting sinusoidal functions to the data. This approach allowed us to look for both periodical and non-stationary variations of the data.

## Results

### Layouts Built and the Choice of the Best one

**Figure 1:**
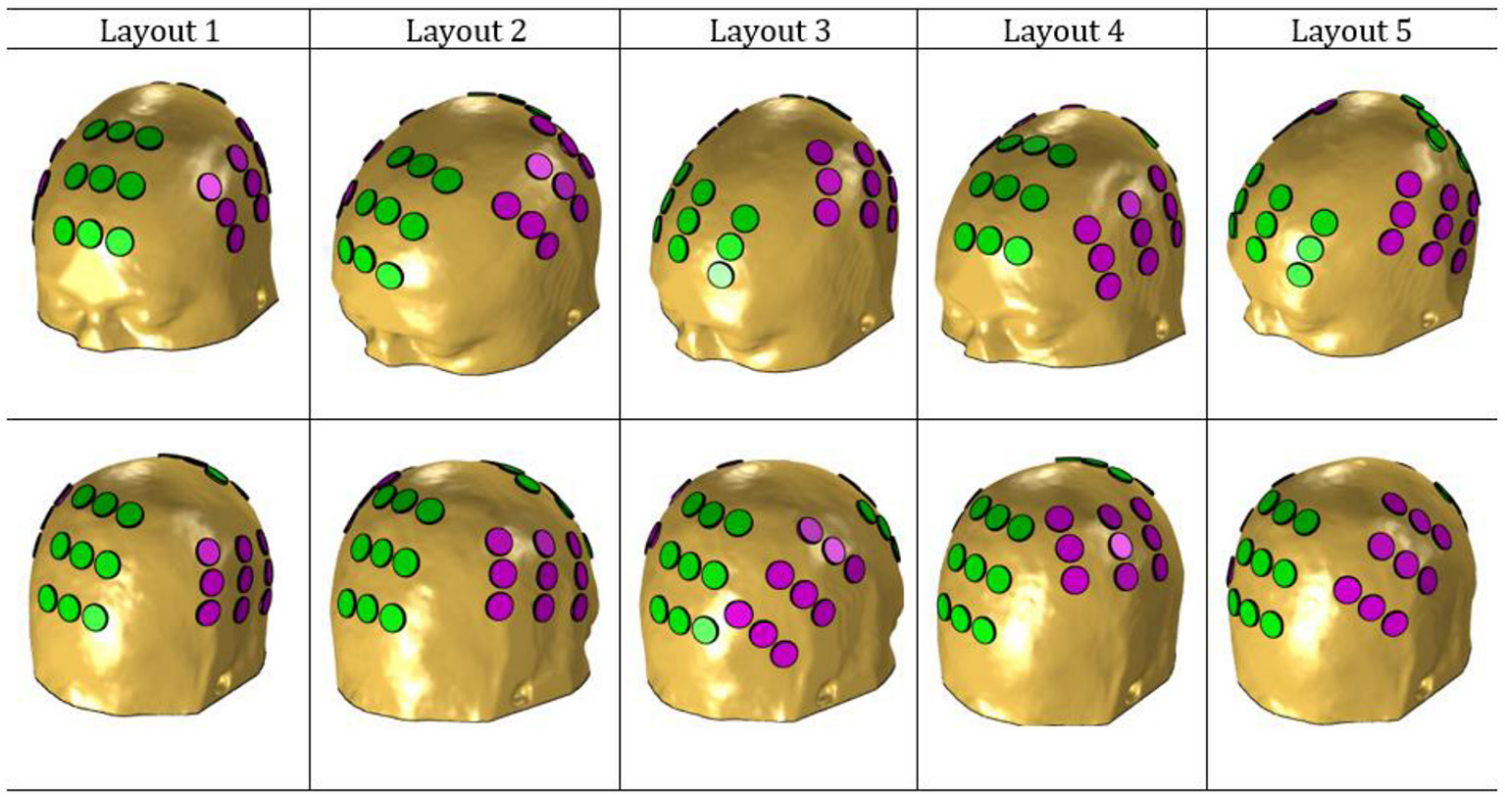
NovoTAL layouts used in this work. The anterior-posterior pair is colored in green and the left-right in magenta.

Figure 1 depicts the five layouts built. The anterior-posterior pair is colored in green, whereas the left-right is colored in magenta.

The values presented in Table 2 show the average EF intensity in the tumor when 900 mA of current were injected into the AP and LR pairs. According to these data, layout 3 was the one that induced the highest electric field in the tumor in the AP direction (0.93 V/cm), whereas layout 4 was the best one for this tumor location when current was injected in the LR pair (1.59 V/cm). The highest average EF was induced by layout 2 (1.23 V/cm) and thus that would be the best choice for this tumor position. At the opposite end, layout 5 would be the worst option with an average value 6% lower (1.16 V/cm).

**Table 2:** Average EF intensity in the tumor when 900 mA were injected into the AP (second column) and into the LR (third column) pairs.

**Layout**	**EF AP (V/cm)**	**EF LR (V/cm)**	**Average EF (V/cm)**
1	0.84	1.57	1.21
2	0.90	1.55	1.23
3	0.93	1.41	1.17
4	0.76	1.59	1.18
5	0.76	1.56	1.16

*The average EF intensity (fourth column) is the criterion typically used to choose the best layout*.

The impedance varied between 59 and 61 Ω in the AP direction, and between 46 and 48 Ω in the LR direction.

### Accounting for the Temperature

**Figure 2:**
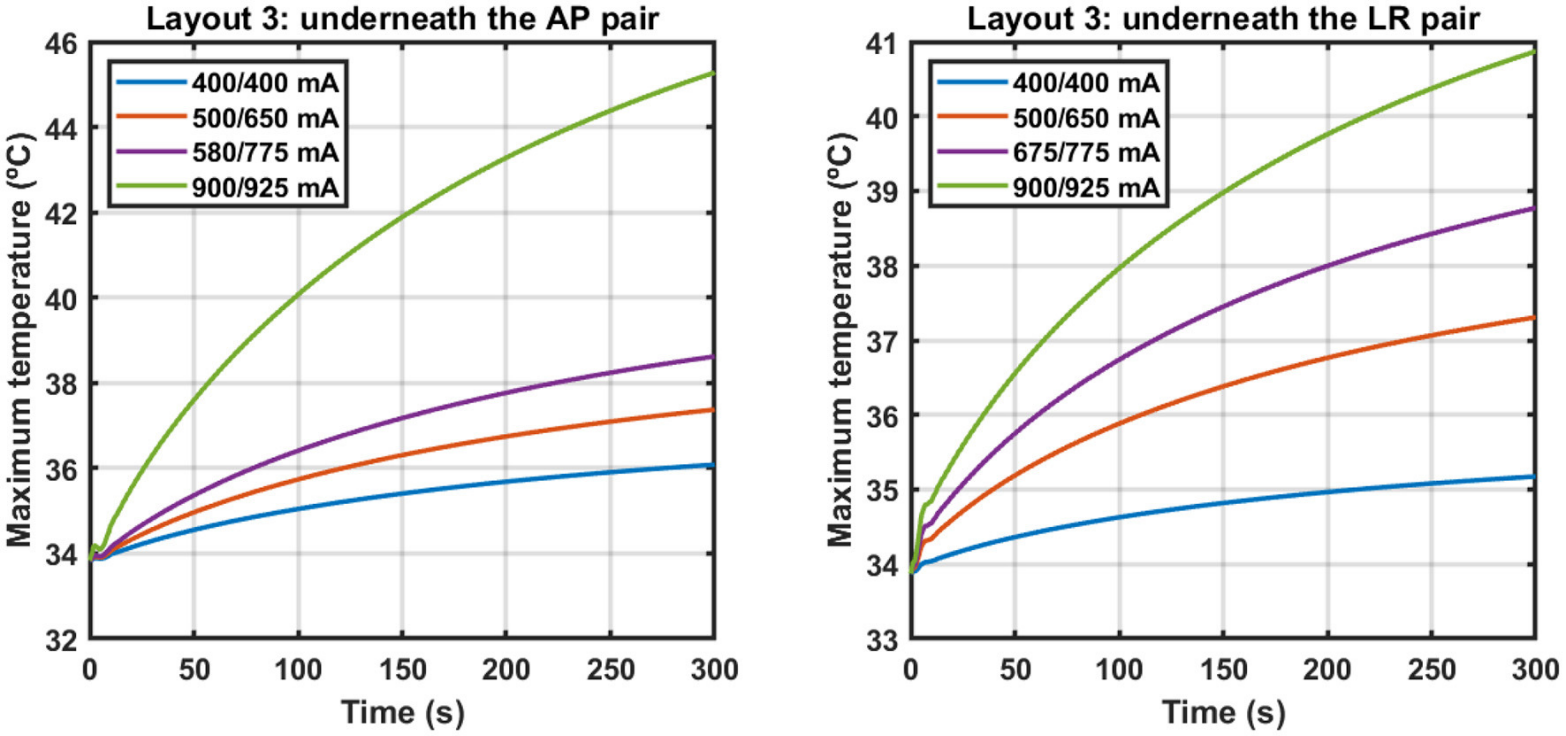
Variation of the maximum temperature on the scalp underneath the AP (**left**) and LR (**right**) pairs when different sets of current were injected using layout 3. Each set is identified by a combination of two values, where the first one corresponds to the current injected into the AP pair and the second one to the current injected into the LR pair. Current was injected alternately with a switching time of one second. The y-axes are different for each plot.

Following the approach described in Subsection How to Account for the Temperature, we investigated how much current could be injected into each pair when information about the temperature was also added to the simulations during treatment planning. Figure 2 shows the variation of the maximum temperature on the scalp underneath each pair for different sets of current injected when layout 3 was used.

In Table 3, the values of the fitted coefficients *C*_1_, *C*_2_ and *C*_3_ are presented after fitting the data depicted above. For each curve the value of the adjusted-R^2^ is also given.

**Table 3:** Values of the fitted coefficients for the different sets of current injected using layout 3.

**Injected current (mA) AP/LR**	**Pair**	**C_1_ (**°**C)**	**C_2_ (s)**	**C_3_ (**°**C)**	**T^max^ (**°**C)**	**Adjusted-R^2^**
400/400	AP	2.7	169	33.9	36.6	0.9995
	LR	1.4	155	33.4	34.8	0.9985
500/650	AP	4.2	170	33.9	38.1	0.9995
	LR	3.6	154	34.1	37.7	0.9983
580/775	AP	5.7	170	33.9	39.5	0.9995
	LR	5.2	155	34.3	39.5	0.9983
900/925	AP	13.3	166	34.0	47.3	0.9994
	LR	7.4	156	34.4	41.8	0.9983

These data indicate that to induce a maximum steady-state temperature of 39.5 °C on the surface of the scalp underneath both pairs simultaneously using layout 3, it is necessary to inject 580 mA into the AP pair and 775 mA into the LR pair with a switching time of 1 s. This represents a reduction of 36% in the first and of 14% in the second compared to when 900 mA were injected. This led to a decrease of the average EF in the tumor of 22% to 0.91 V/cm.

Following the same rationale, we quantified how much current could be injected into each pair using the other layouts. After these simulations were performed, we calculated the average EF in the tumor for those currents. The results are presented in Table 4.

**Table 4:** Amount of current injected into each pair and each layout to induce a maximum steady-state temperature of 39.5 °C on the scalp, or as close as possible to it, underneath both pairs simultaneously.

**Layout**	**I (mA) AP**	**I (mA) LR**	**EF (V/cm)**
1	580 (−36%)	850 (−6%)	1.01 (−17%)
2	580 (−36%)	650 (−26%)	0.85 (−31%)
3	580 (−36%)	775 (−14%)	0.91 (−22%)
4	550 (−39%)	800 (−11%)	0.94 (−20%)
5	565 (−37%)	800 (−11%)	0.93 (−20%)

*Current was injected alternately into each pair with a switching time of 1 s. In parentheses, the relative decrease compared to the values obtained when 900 mA were injected into each pair are presented*.

On average, the current injected into the AP pair decreased by 37% (range: 36–39%), whereas in the LR pair this value was only 14% (6–28%). More current could be injected into the latter as the impedance of the head was lower in that direction. Inevitably, the reduction in these values was accompanied by a decrease in the average EF induced in the tumor, which was reduced by 22% on average (17–31%).

**Figure 3:**
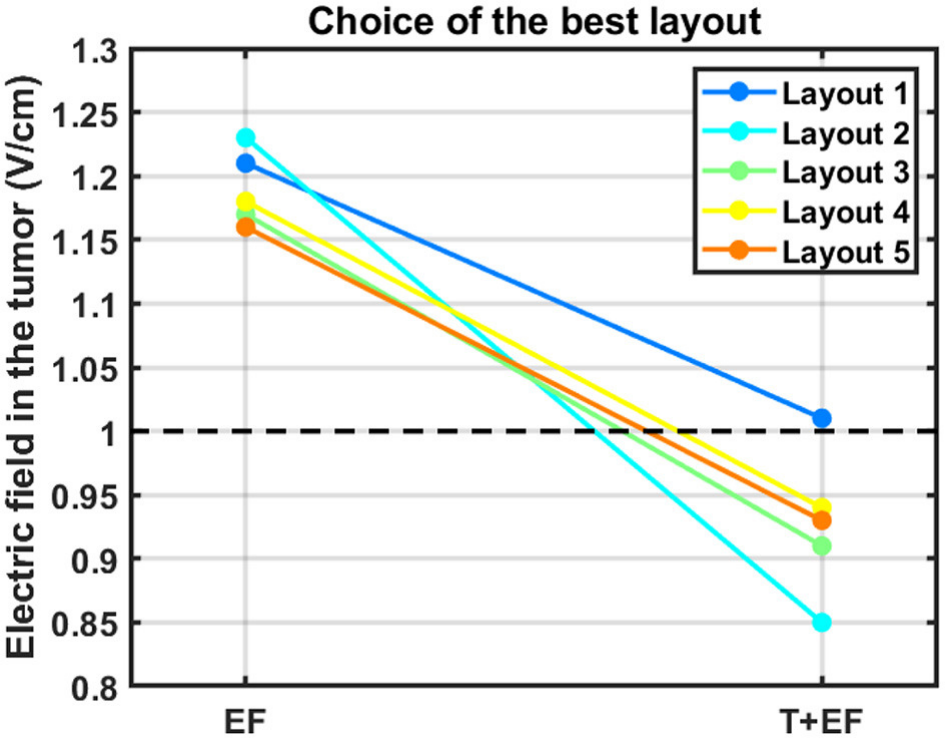
Impact of accounting for the temperature in the choice of the best layout to use. The average electric field intensity in the tumor decreased when this additional parameter was considered (T+EF) compared to when 900 mA were injected in both pairs (EF). The black dashed line represents the therapeutic threshold of 1 V/cm.

Figure 3 depicts a qualitatively comparison of how the choice of the best layout would change if the temperature was also considered.

**Figure 4:**
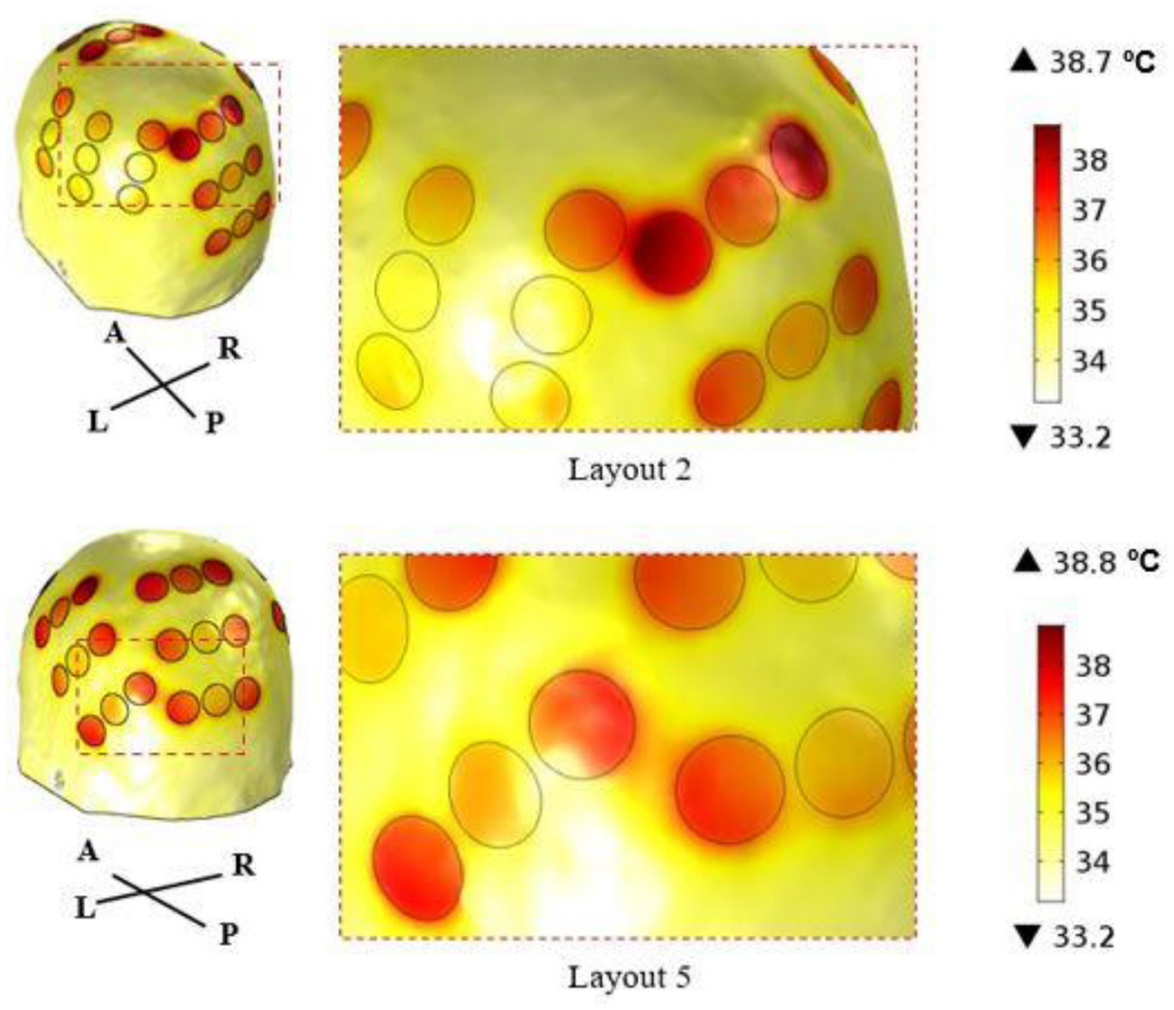
Temperature distribution on the surface of the scalp at the end of the simulation (t = 5 min) using layout 2 (above) and layout 5 (below). The circles represent the interface between the scalp and the gel, and they correspond to the regions where the maximum temperature was reached. In layout 2, the shortest distance between transducers of different arrays was 3 mm, which created a common temperature hotspot in that region. In layout 5, this distance was 8 mm, which was enough to avoid its appearance.

According to this figure, the best layout is now layout 1, which induced an average EF of 1.01 V/cm in the tumor. At the opposite end, layout 2, which was the best one when 900 mA were injected into each pair, is now the least favorable option with an induced EF of 0.85 V/cm. This layout was the only one in which it was not possible to induce 39.5 °C underneath both pairs simultaneously. Underneath the AP arrays the maximum steady-state temperature was the desired value, but underneath LR's it was only 38.3 °C. If more current was injected into the LR pair the temperature of the scalp underneath the AP's would also increase and surpass the 39.5 °C. This occurred because one transducer of the left array was only 3 mm apart from one of the posterior array and thus a temperature hotspot occurred (Figure 4). Consequently, the EF decreased more for this layout compared to the other four when the temperature was also considered. For comparison, the temperature distribution at the end of the simulation for layout 5 is also presented. In this layout, the shortest distance between transducers of different pairs was 8 mm, which was not enough to cause a visible temperature hotspot. In the remaining three layouts this distance was also 8 mm or higher.

### Analysis of the Log Files: Variation of the Impedance Values

**Figure 5:**
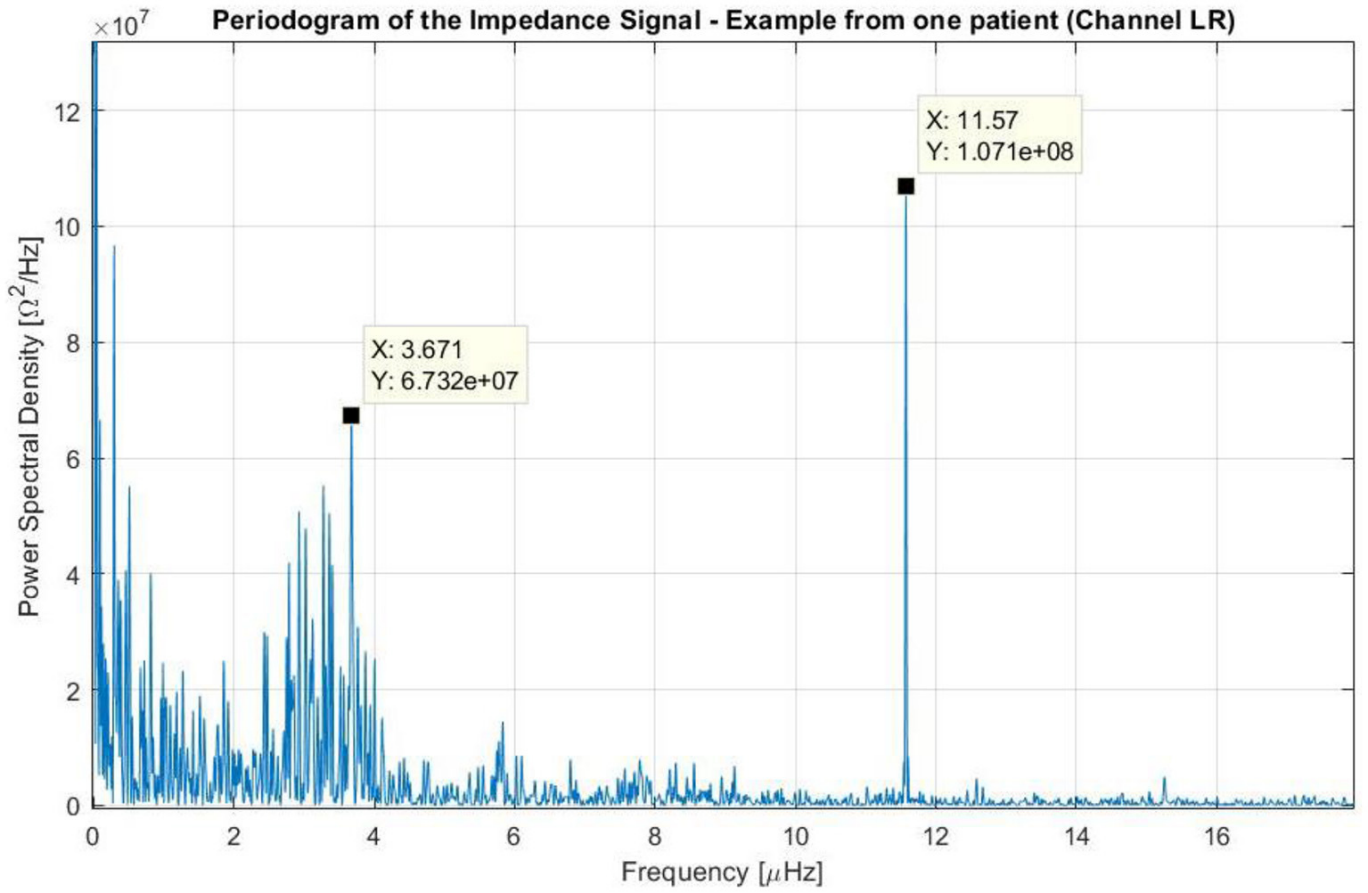
Periodogram of the impedance using the data of one patient treated with TTFields who participated in the EF-14 clinical trial. The variation of the impedance throughout treatment was caused by three main factors: (1) one that occurred at frequencies near the 0 Hz (i.e., long-term variations); (2) one that occurred at frequencies around 3.7 μ*Hz* (which corresponds to a periodicity of around 3 days); and (3) one that occurred at a frequency around 11.6 μ*Hz* (24 h).

The study of the log files from the 340 patients that participated in the EF-14 trial indicated that the average impedance was 78.5 Ω in the AP direction (standard deviation: 12.7 Ω) and 69.0 Ω in the LR direction (standard deviation: 10.4 Ω). The resulting spectra obtained using the Lomb-Scargle method were similar for the majority of patients, with 3 main frequencies. An example of these data is presented for one patient and for the LR pair in Figure 5.

In this figure, the peaks near the 0 Hz correspond to non-stationary variations of the impedance with periods longer than the course of the treatment. These variations with no systematic patterns were observed in all patients, and describe long-term changes (e.g., slow linear increase of the impedance during the course of the whole treatment).

A second peak of interest can be seen around 3.7 μ*Hz*, which corresponds to a time-period of 3 days. This peak has a low amplitude and a wide band. In other patients, this peak can be seen in both channels, only on one channel or in neither of them, and it corresponds to one cycle every 2–4 days (in most cases 3 days). From observing the signal over time, we could deduce that these periods are usually delimited by intervals in which the device has been disconnected for few hours according to the log files. Within each cycle, the impedance increases constantly, reaching values up to 55% higher than the baseline. After the device is disconnected for some time and when treatment is resumed, the impedance drops to its lowest value giving rise to a new period.

**Figure 6:**
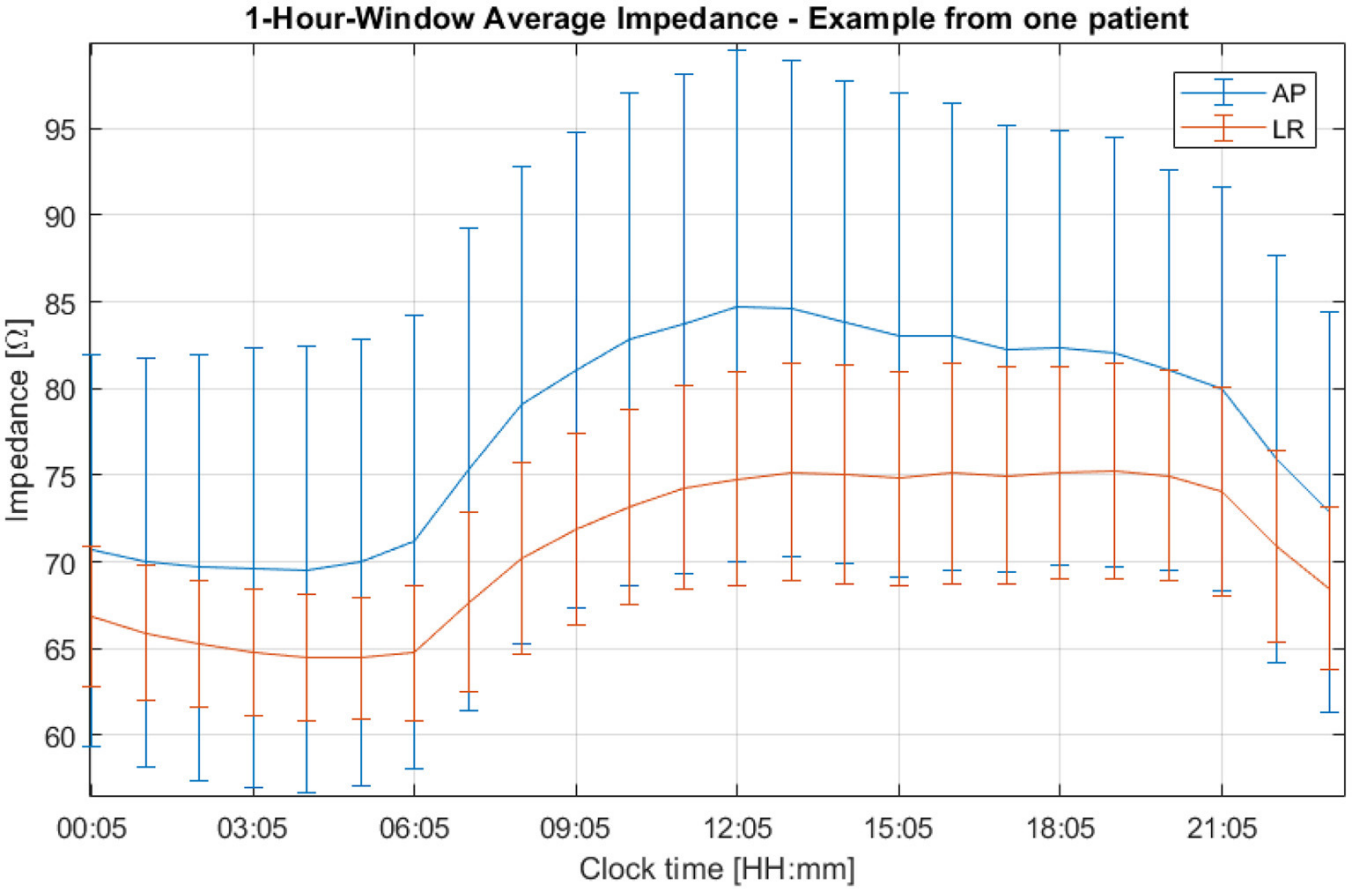
One-hour averaged impedance as a function of the clock time for the AP (in blue) and LR (in orange) array pairs of one patient who participated in the EF-14 clinical trial. The vertical bars are representative of the variability of the data throughout treatment.

A third, more intense and narrower peak can be seen in the previous figure at 11.6 μ*Hz*, which corresponds to a period of 24 h. This cycle can be explained by the one-day variations depicted in Figure 6. In this plot, the average impedance seen during treatment are presented as a function of the clock time for the same patient and for both channels. In most cases, during the nighttime the impedance decreases and stays 5–20% lower than during the daytime. While the pattern is present in both AP and LR channels, the amplitudes for each one of them can be different and usually they are higher in the AP direction than in the LR's, as the head is more resistive in that direction. Contrary to the second peak presented in Figure 5, which was not observed in all patients, this 24-hour variation of the impedance values was common to all of them.

## Discussion

### The Best Layout Based Only on the EF

The layout that induced the highest EF in the AP direction was layout 3 which is explained mainly by how the anterior array was placed on the head. In this layout, this array was oriented horizontally, and it was more centered above the tumor compared to most of the remaining layouts. As the posterior array was also in the most favorable position, this layout yielded the highest EF in this direction. Even though the average EF did not surpass the therapeutic threshold of 1 V/cm in the AP direction for any layout, there were some regions of the active tumor that reached this value. The percentage of the active tumor volume above 1 V/cm (ATV1) was 37% for layout 3. As the tumor was placed close to the right array, the EF intensity was higher in the LR direction compared to AP's. The best LR pair was the one used in layout 4, which induced an average value of 1.59 V/cm. However, in terms of ATV1 it only yielded 86%, a value lower than the 93% obtained using layouts 1 and 2. Overall, in terms of average EF strength, all layouts induced very similar values, which indicates that the NovoTAL system produces very similar solutions for treatment. The best option was layout 2 with 1.23 V/cm, whereas the least favorable one was layout 5 with 1.16 V/cm.

### The Relevance of Accounting for the Temperature

The data presented in Figure 2 and in Table 3 indicate that an exponential function accurately describes the temperature variations when TTFields are applied. Based on the value of *C*_2_ (around 2.7 min, Table 3), at end of the simulation (t = 5 min) the temperature rises due to the application of TTFields had already reached 84% of its steady-state value. Ideally, more time would be simulated but that would require a longer computational time. Thus, a trade-off had to be reached to obtain results in practical time.

In Table 4, the values of current that led to a maximum steady-state temperature of 39.5 °C on the scalp underneath each array are presented. Clearly, it was not possible to inject 900 mA of current in any pair regardless of the layout used. This seems to indicate that the results obtained following the approach typically used in the literature might be overestimating the EF in the tumor. As the criterion followed in this work was to maximize the current injected into the AP pair first, there was an almost uniform decrease in the values seen for the current injected into this pair. The range seen was between 550 and 580 mA, which translates into an average decrease of 37% compared to the 900 mA. As current was reduced by practically the same amount regardless of the layout, this might be an indication that the current injected is more sensitive to the head model than to the position of the arrays.

In the LR pair, this decrease was lower as the impedance of the head in that direction was also lower. On average, the current injected was reduced by only 14%, but the range of variation was higher: 650–850 mA. This wider variation is related with the presence of the temperature hotspot shown in Figure 4. In layout 2, two transducers of different pairs were only 3 mm apart, which created a common temperature hotspot that limited how much current was injected into the LR pair. Thus, the maximum steady-state temperature predicted underneath the left array was only 38.3 °C. In the remaining 4 layouts, the minimum distance between transducers was at least 8 mm, which was enough to avoid the occurrence of a visible hotspot. However, there might still be some influence of the current injected into one pair in increasing the temperature of the scalp underneath the complementary pair. In layouts 3, 4 and 5, the current injected into the LR pair had to be reduced by around 12% (range: 11–14%). In these three cases, the posterior array was close either to the left (layout 5) or to the right (layouts 3 and 4) arrays. In layout 1, in which all arrays were the most separated apart this reduction was the lowest: 6%.

The importance of adding information about the temperature can be seen in Figure 3. In general, accounting for this additional factor increases the range of values obtained for the average EF in the tumor, which allows to differentiate more clearly between layouts. This range increased from 6%, when 900 mA were injected, to 16%. One of the most important conclusions that could be drawn from this work is also highlighted in Figure 3. The best layout when the temperature was not considered, layout 2, was the least favorable option for treatment when this additional factor was taken into consideration. Based on these data, we suggest a minimum distance of 1 cm between arrays, which ensures that the target temperature of 39.5 °C can be reached underneath both pairs simultaneously. Ideally, temperature-wise, the arrays should be placed as far as possible from each other to reduce the contribution that one pair has in increasing scalp's temperature underneath the other. This approach represents one indirect way to account for the contribution of the temperature during TTFields treatment planning. As seen in Figure 3 (excluding data from layout 2), the ranking of the best layout is not affected by this additional parameter provided that the arrays are at least 8 mm apart from each other.

### Analysis of the Impedance Data

As the impedance varies throughout treatment, the amount of current necessary to induce a maximum temperature of 39.5 °C on the scalp might vary considerably. Thus, the methodology discussed in Subsection How to Account for the Temperature to predict how much current could be injected into each pair is prone to errors as the impedance of the model was assumed to be constant. Even though that value was close to what was seen in the log files of one patient treated with TTFields for the same electric parameters as the ones used by Ballo et al. (2019), a more extensive analysis of other patients' data showed a more complex intra- and inter-subject variability of these values.

The spectral analysis presented in Figure 5 allowed us to investigate and understand better the variations of the impedance and their periodicity. We concluded that there were three main contributions to these changes. The first one, that could be observed at very low frequencies, is related with slow increases or decreases in the impedance throughout treatment. The second peak, whose periodicity was around 3 days, is related with replacement of the arrays and it plays a role in the value of the contact impedance at the gel-scalp interface. Although we do not have any data indicating when EF-14 participants replaced the transducer arrays during treatment, patients are advised to do so at least every 3 days. After removing the arrays, they shave their heads and usually allow for some time to let the skin breathe before applying new ones, generally after taking a shower. Thus, it is highly probable that this peak is related to array replacement and shaving procedure. As patients are instructed on how to replace the arrays by themselves, there is no way to guarantee that this is done exactly every three days at the same time, which could explain the wider range seen for this peak. The fact that these impedance variations can be observed in either both channels, only one, or neither of them, may indicate that the hair growth is a key factor on the impedance. Furthermore, there are different dermatologic adverse events that occur in patients treated with TTFields. As discussed by Lacouture et al. (2020), these are a consequence of mechanical, thermal, chemical, and moisture-related stresses that result from a prolonged contact between the scalp and the transducer arrays/medical tape applied to the same area of the patient's head. All these effects can contribute significantly to changes in the dielectric properties of the scalp and consequently to variations in the contact impedance. Another main factor that affects this value are the mechanical perturbations on the attachment of the arrays to the scalp. A bad contact between these two (e.g., due to a partial detachment of the arrays) can increase the impedance and thus contribute with noise to the values registered in the log files. However, it is not reasonable to believe that it can affect the periodical pattern represented by the third peak in Figure 5.

In general, the impedance reaches its lowest value at night and its highest during the day (Figure 6). Impedance variations in humans have been studied by several authors (e.g., Boucsein, 2012). Most of the research published in the literature focuses on measurements in the fingers, hands, and forearms, usually with very low power (around 1 W, whereas for TTFields the power is around 50 W) and a variety of frequencies, as well as DC. Circadian variations have been studied on several occasions, although most of the results showed an opposite behavior to the one reported here when measuring the impedance at the arms: during the night the recorded impedance was higher (Koumans et al., 1968). With regard to the circadian variations, the pressure applied to the head by the pillow during the sleep phase could indeed improve the electrode attachment and explain the decrease in the impedance. However, as we see that both channels follow the circadian pattern constantly, the added pressure cannot fully explain this behavior, as it may be applied only in one of the four arrays at a time. During the night, patients are more still, which could also improve the contact between the arrays and the head and thus decrease the impedance registered by the Optune device. The impact of other factors such as the temperature and the sweat might also play a significant role in these variations, although an analysis of its impact needs to take into consideration possible chemical interactions with the gel and consequent changes in its electric conductivity.

### Impact of These Conclusions in Computational Results and Future Work

The accuracy of the conclusions drawn from computational studies is intrinsically related to the use of a correct set of dielectric parameters of tissues. One way to validate the model and to ensure that the results obtained from it are reasonably accurate is by comparing its impedance with the impedance seen in the log files of patients. One of the main challenges in doing this is related with the inter-subject variability of these values. The impedance of the model was around 60 Ω in the AP direction and 47 Ω in the LR's for the electric parameters presented in Table 1. This range of values was close to what was seen in the log files of the patient used to investigate how to account for the temperature during treatment planning. However, the analysis of the impedance of the subgroup of patients that participated in the EF-14 clinical trial showed that in most cases the head is less conductive as the impedance was, on average, 78.5 Ω in the AP direction (standard deviation: 12.7 Ω), and 69.0 Ω in the LR direction (standard deviation: 10.4 Ω). These values also showed a significant intra-subject variability that depended on three different factors and whose variations makes it more challenging to choose which values to use for the electric parameters in simulations.

One possible approach that might help to improve the accuracy of the results consists in using a technique named water-content based electrical properties tomography (wEPT), as discussed by Wenger et al. (2019). The basis of this technique is that tissue electric conductivity is highly correlated with its water content and by estimating the latter it is possible to predict the former. The methodology followed in this technique consists in acquiring two T1-magnetic resonance (MR) images with different repetition times (TR), calculate the ratio of the signal intensity for each voxel, and relate that information with the electric properties. Thus, through this technique it is possible to reduce the uncertainty on the electric parameters and consequently improve the accuracy of computational simulations. One of the main challenges in doing this is acquiring more MR data, which is not cost-effective nor practical to do for every patient. Furthermore, this approach might only allow to decrease the uncertainty on the electric parameters to some extent as the variations attributed to array replacement and circadian rhythm are more difficult to control.

Based on the impedance variations discussed above, the scalp is the tissue whose electric conductivity might vary the most as it is the one in direct contact with the arrays and thus most likely to be affected by the three-day cycle variations. As the scalp is also the tissue that heats up the most during treatment and that the Joule effect is the main source of heating in this tissue when TTFields are applied (Gentilal and Miranda, 2020), the importance of an accurate estimation of the electric conductivity of this tissue is crucial. Future studies that investigate the impedance variations should also consider the fact that the electric properties might change with the temperature. There are not many studies in the literature that addressed how to accurately account for these variations for the conditions in which TTFields are applied. These include measurements for electric fields with a frequency between 100 and 500 kHz, applied continuously for at least 18 hours per day and for a temperature range between 20 and 40 °C. As discussed by McIntosh and Anderson (2010) and by Rossmanna and Haemmerich (2014) one possible way to account for this dependence is assuming that the physical parameters vary linearly with the temperature, although the coefficients of this relationship still need to be investigated. Accounting for the variations of the impedance that occur during treatment might also change the conclusions in terms of how much current can be injected and on the minimum distance between arrays to avoid the occurrence of temperature hotspots. If the impedance of the scalp increases less can current can be injected into the arrays to limit scalp's temperature to a maximum of 39.5 °C. In this context, additional future work might include performing a similar thermal analysis to the one presented here but using different values for the dielectric properties. Likewise, using a different set of thermal parameters might also lead to different conclusions in terms of how much current can be injected into each array pair. It was shown that the blood perfusion in the human forearm skin can increase as much as 15 times during heating (Song et al., 1990). As the scalp cools down mainly through conduction and blood perfusion when TTFields are applied (Gentilal and Miranda, 2020), considering these variations could lead to a more optimistic prediction of the EF in the tumor as the scalp would cool down more efficiently.

The results presented in this work highlight that the changes that occur in terms of temperature and impedance play a significant role in the study of Tumor Treating Fields and its effectiveness and thus they should be considered in future studies.

## Data Availability Statement

The original contributions presented in the study are included in the article/supplementary material, further inquiries can be directed to the corresponding author.

## Author Contributions

NG: conceived and performed the modeling studies, analyzed and interpreted the computational data, and wrote part of the original paper. EA: analyzed and interpreted the impedance data and wrote part of the original paper. AN: produced the layouts in the NovoTAL system and offered critical revisions. IB and YT: offered critical revisions. TM: analyzed and interpreted the impedance data and offered critical revisions. PCM: conceived the modeling studies, analyzed and interpreted the computational data, reviewed the writing of the original draft, and offered critical revisions. All authors reviewed, read, and approved the final manuscript.

## Funding

Instituto de Biofísica e Engenharia Biomédica (IBEB) is supported by Fundação para a Ciência e Tecnologia (FCT), Portugal, under grant no. UIDB/00645/2020.

## Conflict of Interest

NG holds a PhD grant funded by a research agreement with Novocure. EA, AN, TM, IB, and YT are employed by and hold stock in Novocure. PCM has a research agreement with Novocure.

## Publisher's Note

All claims expressed in this article are solely those of the authors and do not necessarily represent those of their affiliated organizations, or those of the publisher, the editors and the reviewers. Any product that may be evaluated in this article, or claim that may be made by its manufacturer, is not guaranteed or endorsed by the publisher.
